# Host cyclophilin A facilitates SARS-CoV-2 infection by binding and stabilizing spike on virions

**DOI:** 10.1038/s41392-023-01719-7

**Published:** 2023-12-14

**Authors:** Xiangpeng Sheng, Fang Zhu, Hong Peng, Fan Yang, Yi Yang, Cong Yang, Zhen Wang, Wei Chen, Deyin Guo, Ronggui Hu

**Affiliations:** 1 State Key Laboratory of Molecular Biology, Center for Excellence in Molecular Cell Science, Shanghai Institute of Biochemistry and Cell Biology, University of Chinese Academy of Sciences, Chinese Academy of Sciences, Shanghai, China; 2 MOE Key Laboratory of Tropical Disease Control, Centre for Infection and Immunity Study, School of Medicine, Sun Yat-sen University, Shenzhen, China; 3 ICU, Longhua Hospital Shanghai University of Traditional Chinese Medicine, Shanghai, China

**Keywords:** Microbiology, Biochemistry

**Dear Editor**,

The novel coronavirus SARS-CoV-2 rapidly evolutes to increase its infectivity and transmissibility, facilitates its immune escape, impairs vaccine efficacy, and currently causes repeated infections in human, which is mainly determined by the accumulated mutations in spike (S).^1^ Transmembrane S is a heavily glycosylated protein on the surface of the virion as homotrimer, responsible for binding to human angiotensin-converting enzyme 2 (hACE2) receptor.^2^ While the architecture of S trimers has been well studied, how the stability of S is regulated by potential host factors remains unknown.

To identify potential interaction partners of S in host cells, we performed a human genome-scale yeast two-hybrid (Y2H) screen with S1 as a bait against an ORFeome collection (~ 15000 ORFs). Several candidates were identified in the Y2H assay after DNA sequencing of yeast clones growing from SD4 plates (Supplementary Fig. S1a). Purified GST-fused candidates were subjected to a GST pull-down assay with the cell lysates of HEK293T expressing S (Fig. 1a). While other GST fusion proteins did not capture S and S1, human cyclophilin A (CypA or PPIA) showed a high capacity to interact with S and S1. Moreover, the yeast strain simultaneously carrying S (or S1) and CypA, but not the strains that only express one of them, had the ability to grow on SD4 plates, verifying the S-CypA interaction in yeast cells (Fig. 1b).

**Fig. 1:**
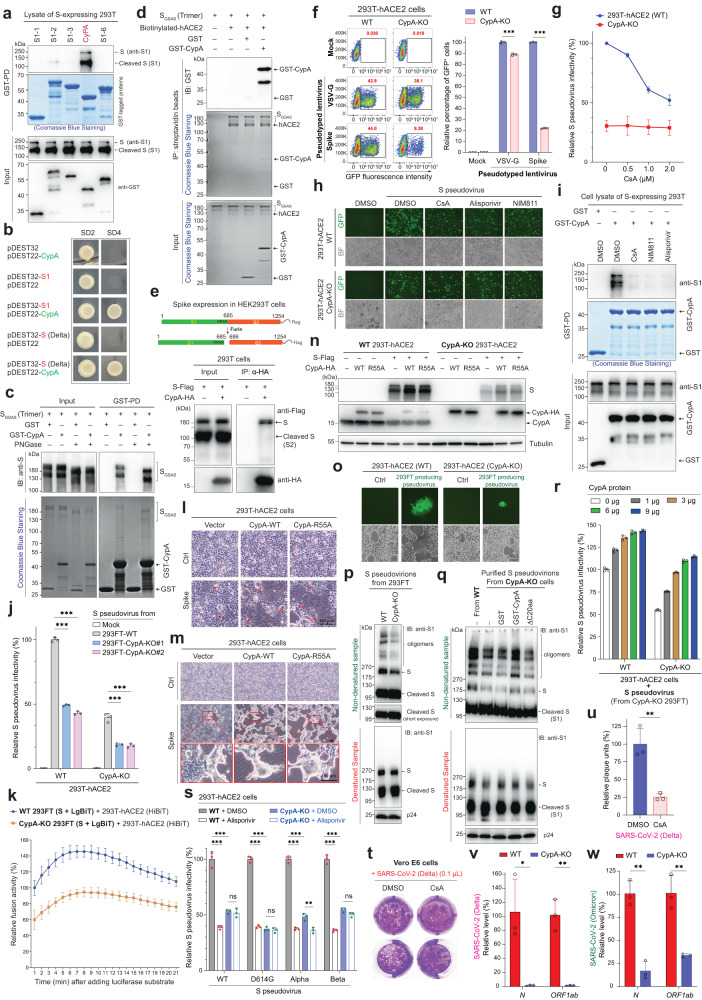
Host CypA facilitates SARS-CoV-2 infection by interacting with and regulating spike on the virions. **a** SARS-CoV-2 spike was found to interact with human CypA. 293 T cells expressing S were lysed in Co-IP buffer and subjected to a GST pull-down (PD) assay with recombinant GST-CypA. Samples were analyzed by anti-GST or anti-S1 immunoblotting (IB). **b** S1 or S binds to human CypA in the Y2H system. Plasmids that express S1 (or S) and/or CypA were transfected to yeast cells, and the growth of yeast on SD2 and SD4 agar plates was estimated after about two weeks. **c** Trimeric S_GSAS_ protein interacts with CypA in vitro. Non-cleaved S_GSAS_ mutant (substitution of RRAR (682-685aa) by GSAS) was purified from 293 T primarily in trimeric forms. S_GSAS_ trimers was subjected to GST- or GST-CypA-based PD assay supplemented with or without PNGase, at 4 °C overnight. **d** S-hACE2 complex can recruit CypA. Trimeric S_GSAS_ was incubated with biotinylated hACE2 and GST or GST-CypA, and then Co-IP with streptavidin beads was conducted. **e** CypA prefers full-length S, but not its cleaved form (S2). 293 T cells transfected with S and CypA-HA plasmids were lysed in Co-IP buffer and subjected to anti-HA co-IP and subsequential western blots. **f** CypA deficiency robustly blocks S pseudovirus infection. WT or CypA-KO 293T-hACE2 cells were incubated with S pseudovirus or VSV-G pseudovirus. After 24 h, cells were analyzed by flow cytometry. Shown in the left panel are flow cytometric plots with the proportions of GFP-positive cells. A comparison of percentages of GFP-positive cells are present in the right panel (*n* = 3). **g** Removal of CypA in targeted cells eliminates the inhibitory effect of CsA on viral infection. WT or CypA-KO 293T-hACE2 was infected by S pseudovirus with different concentrations of CsA. GFP-positive cells were detected by flow cytometry and relative infectivity was calculated (*n* = 3). **h** CsA and its derivatives inhibit S pseudovirus infection by targeting CypA. WT or CypA-KO 293T-hACE2 cells were incubated with equal virions and indicated inhibitors (1 μM) for 24 h. GFP-fluorescent images and bright field (BF) images were taken by microscopy. Scale bars, 50 μm. **i** CsA and its derivatives block S recruitment to CypA. Cell lysates of S-expressing 293 T cells, GSH beads, GST-CypA, and CypA inhibitors (with final 1 μM) were incubated in PD buffer for 2 h. GSH beads were then collected, washed, and analyzed by Coomassie blue staining of SDS-PAGE gel or anti-S1 IB. **j** Deficiency of CypA in packaging cells impairs viral infectivity. WT or CypA-KO 293T-hACE2 cells were infected by viruses produced from one WT or two CypA-KO 293FT packaging cell lines for 24 h. Cells were analyzed by flow cytometry (*n* = 3). **k** CypA depletion in S-expressing cells attenuates S/hACE2-mediated cell–cell fusion. 293T-hACE2 cells expressing HiBiT were incubated with WT or CypA-KO 293FT cells expressing S and LgBiT. 5 h later, substrate furimazine was added to cells and luminescence intensities were detected (*n* = 3). **l** Overexpression of CypA exacerbates S-mediated cell-cell fusion in 293T-hACE2. Empty vector or S-expressing plasmid was co-transfected with the plasmid encoding WT or R55A CypA in 293T-hACE2 cells for 18 h. Cell fusion events were analyzed by microscopy. Shown are the bright field (BF) images (scale bars, 100 μm). Red arrowheads mark cell-cell fusion. **m** CypA dramatically promotes the formation of S-mediated giant syncytia in 293T-hACE2. Cells were transfected with S and CypA or its R55A mutant for about 30 h. BF images were taken by microscopy. Scale bars, 100 μm. **n** CypA expression in host cells stabilizes S protein. Plasmids expressing S and/or CypA-HA (or CypA-R55A-HA) were co-transfected into WT or CypA-KO 293T-hACE2 for 24 h. Samples were collected and subjected to anti-S1, anti-CypA, or anti-Tubulin IB analysis. **o** Cell fusion-mediated viral transmission in 293T-hACE2 is blocked by CypA deficiency. 293FT cells producing S pseudovirus were co-cultured with WT or CypA-KO 293T-hACE2 cells for 24 h, and then images were acquired by microscopy. Scale bars, 50 μm. **p** Deficiency of CypA in the packaging cells decreases S oligomers on the virions. S pseudovirus particles produced from WT or CypA-KO 293FT cells were lysed to prepare non-denatured and denatured samples for anti-S1 western blot. **q** CypA facilitates S oligomers formation on virions. Purified S pseudovirions from CypA-KO cells was incubated with GST, GST-CypA, or GST-CypA-△C20aa at 37 °C for one hour. Non-denatured and denatured samples of virions were analyzed by anti-S1 or anti-p24 western blot. **r** Incubation with recombinant CypA protein increases the infectivity of S pseudovirus. Purified S pseudovirions from CypA-KO cells was incubated with different amounts of recombinant CypA protein at 37 °C for one hour. These S pseudovirions were then utilized to infect WT or CypA-KO 293T-hACE2 for 24 h. Cells were analyzed by flow cytometry (*n* = 3). **s** Alisporivir inhibits the infection of SARS-CoV-2 variants (D614G, Alpha, or Beta) pseudovirus by targeting CypA. WT or CypA-KO 293T-hACE2 treated with or without alisporivir (1 μM) was infected by different pseudovirus and analyzed by flow cytometry (*n* = 3). **t**, **u** CsA strongly restricts plaque formation of SARS-CoV-2 Delta. Vero E6 cells treated with or without CsA were incubated with 0.1 μL SARS-CoV-2 Delta and seeded into the 12-well plates. When the plaques were formed, cells were fixed and stained with 0.5% (w/v) crystal violet. Shown in **t** are images, and shown in **u** is the quantitative data of plaque-forming units (*n* = 3). **v**, **w** Knockout of CypA in 293T-hACE2 cells blocks infection of SARS-CoV-2 Delta or Omicron. WT and CypA-KO 293T-hACE2 cells were infected by Delta (**v**) or omicron (**w**) variant. 48 h later, culture supernatant was subjected to qPCR analysis of viral gene *N* or *ORF1ab*. Data are presented as mean ± SD (Student’s *t*-test). *p < 0.05, **p < 0.01, ***p < 0.001, ns: no significance

Being a cytoplasmic or secretory protein, CypA has isomerase activity and chaperone-like function to enhance protein folding and trafficking, which controls many biological processes and contributes to diverse viral infections, including HIV, HBV, and some coronaviruses. Recently, cyclosporine (CsA), a cyclic peptide inhibitor of CypA, and its derivatives were found to be efficient against SARS-CoV-2.^3^ However, the mechanism by which CypA inhibitors modulate SARS-CoV-2 infection remains unknown. Therefore, we hypothesize that CypA may facilitate SARS-CoV-2 infection by binding and regulating S protein. A series of assays were then conducted to estimate the interaction between CypA with S. We purified non-cleaved trimeric S mutant (S_GSAS_) from HEK293T cells to perform interaction assays. GST-CypA, but not GST alone, can interact with trimeric S_GSAS_ in vitro or capture S_GSAS_ expressed in cells (Fig. 1c and Supplementary Fig. S1b). Moreover, PNGase F that removes N-linked glycans seemed to promote S recruitment to CypA, suggesting an inhibitory role of S glycosylation on CypA binding (Fig. 1c). Next, we tested if CypA has an impact on the receptor binding of S trimers. As shown, the high affinity between hACE2 and S_GSAS_ can lead to S_GSAS_-hACE2 complex formation in vitro, which cannot be disturbed by CypA (Fig. 1d). More importantly, the complex of S_GSAS_-hACE2 significantly recruited GST-CypA, but not GST. Because hACE2 does not bind to CypA,^4^ S-hACE2 complex probably recruits CypA via S. In human cells, part of wild-type (WT) S was cleaved by host proteases at the S1/S2 site. Notably, a co-immunoprecipitation (Co-IP) result showed that CypA preferentially captured full-length S, but displayed a low affinity to cleaved S (S2) (Fig. 1e), which suggests that CypA may bind to the S1 domain. Indeed, 293T-expressed S1 was readily recruited by CypA (Supplementary Fig. S1c). Coincidentally, during the preparation of this manuscript, a new study also demonstrated a strong interaction between the S1 RBD and CypA.^4^ Therefore, S binds to CypA via its S1 domain.

To investigate the possible impacts of CypA on the viral infection, spike-pseudotyped lentivirus (S pseudovirus) containing a super-folder GFP (sfGFP) reporter was packaged in HEK293FT cells by a three-plasmid system (Supplementary Fig. S2a, b), and used to infect WT or CypA-knockout (KO) 293T-hACE2 cells constructed by CRISPR/Cas9 technology (Supplementary Fig. S2c, d). As shown, the percentage of S pseudovirus-infected population in CypA-KO 293T-hACE2 was several times lower than that in WT (Fig.1f and Supplementary Fig. S2e, f), indicating that CypA deficiency in 293T-hACE2 effectively decreased cell susceptibility to S pseudovirus. However, the knockout of CypA only had a weak effect on the infection of VSV-G pseudovirus (Fig.1f and Supplementary Fig. S2g). Notably, CypA depletion in 293T-hACE2 cells did not decrease ACE2 (Supplementary Fig. S2h). Thus, these results suggest that CypA deficiency impairs the infectivity of S pseudovirus by affecting S function.

CypA is a cellular receptor of CsA, an immunosuppressive drug that was reported to inhibit SARS-CoV-2 infection.^3^ Indeed, CsA exhibited a dose-dependent ability to suppress S pseudovirus infection in 293T-hACE2 cells (Supplementary Fig. S2i-k). Moreover, we found that CsA antiviral activity was eliminated by CypA deficiency in cells (Fig. 1g and Supplementary Fig. S2l), which suggests that CypA expression in host cells is the precondition for the antiviral effect of CsA. Moreover, CsA significantly disrupted S recruitment to the CypA-bound beads in a dose-dependent manner (Supplementary Fig. S2m). We also tested the potential effects of non-immunosuppressive analogs of CsA: NIM811 and alisporivir (Debio-025) (Supplementary Fig. S3a). As expected, NIM811 or alisporivir showed inhibitory effects on the viral infection in CypA-expressing cells, similar to CsA (Fig. 1h and Supplementary Fig. S3b–d). These two analogs of CsA also potently suppressed S recruitment to CypA (Fig. 1i). Although CsA or its analogs block viral infection, they had no effect on the expression of ACE2 or sfGFP (Supplementary Fig. S3e, f). These data indicate that CsA may restrict S pseudovirus infection via disrupting CypA-S interaction.

Because S pseudovirus-packaging 293FT cells also express high-level CypA protein, roles of CypA of the packaging cells on viral infectivity were next investigated. CypA-deficient 293FT cell lines were constructed for pseudovirus production (Supplementary Fig. S4a, b). While WT and CypA-KO 293FT cell lines had comparable transfection efficiencies, S pseudovirus produced from CypA-KO 293FT exhibited much weaker infectivity than that from WT 293FT (Fig. 1j and Supplementary Fig. S4c, d). The infection of S pseudovirus from CypA-KO 293FT was further impaired when the targeted 293T-hACE2 were also CypA-deficient. Moreover, these results were further confirmed by microscopic analysis and western blot (Supplementary Fig. S4e, f). Thus, CypA deficiency in virus-packaging cells impairs the production or infectivity of S pseudovirus.

Next, possible impacts of CypA on S-mediated membrane fusion were examined. We constructed a split NanoLuc-based cell-cell fusion reporter system, in which 293T-hACE2 cells expressing HiBiT were mixed with other cells that overexpress S and LgBiT (Supplementary Fig. S5a). The S-hACE2 interaction on cell surfaces can lead to cell fusion, which allows HiBiT recruitment to LgBiT in the fused cells, forming active luciferase enzymes to generate bioluminescence (Fig. 1k). Deficiency of CypA in S-overexpressing cells attenuated cell fusion-based bioluminescence, indicating that CypA modulates S/hACE2-induced cell–cell fusion. In 293T-hACE2 cells, S overexpression alone induces cell–cell fusion; moreover, simultaneous expression of S and CypA readily exacerbated S-mediated fusion (Fig. 1l and Supplementary Fig. S5b). Because giant syncytia and cell death usually occur after extensive cell fusion, the effects of CypA on syncytia formation were estimated. Consistently, co-expression of CypA strongly promoted the formation of giant syncytia caused by S (Fig. 1m and Supplementary Fig. S5c). We next examined the S protein level and found that it was controlled by CypA expression level (Fig. 1n). While S was decreased in CypA-KO cells, CypA overexpression or rescue increased S protein level. These data suggest that CypA facilitates S-mediated membrane fusion by stabilizing S in mammalian cells.

Cell–cell fusion has been an efficient strategy for coronaviruses to directly infect neighboring cells. To investigate whether CypA may regulate cell-to-cell viral transmission, we employed a dynamic pseudovirus-based cell-cell fusion assay, as described before (Supplementary Fig. S5d).^5^ As shown, initiating from an S pseudovirus-producing cell, more and more neighboring 293T-hACE2 cells fused together and then transferred virions to more surrounding cells, finally forming GFP-positive large syncytia (Fig. 1o and Supplementary Fig. S5e, f). However, CypA deletion in the targeted hACE2-positive cells restricted syncytia formation and virus diffusion. These results, thus, indicate that CypA deficiency may disrupt virus transmission by decreasing S stability.

As loss of CypA isomerase activity did not perturb its role in accelerating S-mediated cell-cell fusion and syncytia formation (Fig. 1l-m and Supplementary Fig. S5b, c), we hypothesized that CypA may function as a chaperone to promote S folding and trimer formation on virions. To prove this, we first purified S pseudovirus, and found that CypA was readily retained in the purified S pseudovirus, but not VSV-G pseudovirus, indicating that the S-containing virions recruit CypA (Supplementary Fig. S6a–c). As shown, the purification process resulted in the complete removal of medium components in the viral supernatant, and the significant enrichment of recombinant virions. S pseudovirions were solubilized in Triton X-100 buffer and then subjected to a GST pull-down assay. By western analysis of S non-denatured or denatured forms according to a literature,^6^ recombinant GST-CypA, but not GST or C-terminal-deleted CypA (△C20), readily precipitated monomeric and oligomeric S from viral lysates (Supplementary Fig. S6d). Moreover, via comparing pseudoviruses produced from WT or CypA-KO 293FT, we found that CypA-KO cells-produced S pseudovirus had fewer S oligomers than that from WT cells (Fig. 1p), suggesting that CypA deficiency impairs S oligomers formation. More importantly, incubation of S pseudovirus with recombinant GST-CypA, but not GST, readily increased S oligomers (Fig. 1q and Supplementary Fig. S6e). Consistently, the CypA-△C20 mutant that lacks S-binding capacity did not promote S oligomerization. Because S functions as trimer on the virion surface, we next explored the role of recombinant CypA on S pseudovirus infection. As shown, incubation with recombinant CypA increased the infectivity of S pseudovirions from CypA-KO 293FT (Fig. 1r and Supplementary Fig. S6f). Thus, these data suggest that CypA can promote SARS-CoV-2 infection via facilitating S trimer formation, which stabilizes S in host cells.

SARS-CoV-2 circulation in human population since December 2019 has evolved many variants of concern (Supplementary Fig. S7a). Depletion or inhibition of CypA in hACE2-expressing 293 T cells significantly suppressed the infection of D614G, Alpha, or Beta pseudovirus, similar to WT S pseudovirus (Fig. 1s). Infection assays with authentic SARS-CoV-2 variants (Delta or Omicron) were further performed to confirm the role of CypA in promoting SARS-CoV-2 infectivity. CsA and its derivates suppressed SARS-CoV-2 Delta infection in Calu-3 and Vero E6 cells (Supplementary Fig. S7b-f). A plague-forming assay revealed that CsA also robustly decreased plague numbers of the SARS-CoV-2 Delta variant (Fig. 1t, u and Supplementary Fig. S7g). Finally, we conducted the authentic SARS-CoV-2 infection assay in WT and CypA-KO 293T-hACE2 cells. Both qPCR and western blot showed that CypA deletion strongly restricted SARS-CoV-2 Delta infection (Fig. 1v and Supplementary Fig. S7h). SARS-CoV-2 Omicron also showed weaker infectivity toward CypA-KO 293T-hACE2 than WT 293T-hACE2 (Fig. 1w). Thus, targeting CypA impairs multiple SARS-CoV-2 variants infection in host cells.

In summary, our study uncovers a novel mechanism that modulates S trimerization and stability. We demonstrate that CypA, a ubiquitously expressed protein in mammals, interacts with SARS-CoV-2 S protein and increases S-mediated membrane fusion and viral infectivity. Targeting CypA by using cyclic peptide inhibitors or deleting *CypA* gene in targeted cells readily prevents the infection of multiple SARS-CoV-2 variants. Moreover, we highlight a novel mechanism that CypA stabilizes S by facilitating S folding and trimer formation. This previously unknown mechanism expands our understanding of SARS-CoV-2 biology and emphasizes CypA as a target for antiviral therapy.

### Supplementary information

Materials and Supplementary Figures

## Data Availability

All data are presented in the main text or the supplementary materials, and can be obtained from the corresponding authors upon reasonable request.
