# Supplementary material for: Host cyclophilin A facilitates SARS-CoV-2 infection by binding and stabilizing spike on virions
Source: Signal Transduct Target Ther. 2023 Dec 14;8:459. doi: 10.1038/s41392-023-01719-7 (PMC10721623; doi:10.1038/s41392-023-01719-7)
Supplement: Supplementary file 1 — Materials and Supplementary Figures [file 41392_2023_1719_MOESM1_ESM.pdf]

Supplementary Materials for

Host cyclophilin A facilitates SARS-CoV-2 infection by binding and stabilizing spike on virions

Xiangpeng Sheng[1,#], Fang Zhu[1,#], Hong Peng[2,#], Fan Yang[1,#], Yi Yang[1,#], Cong Yang[1], Zhen Wang[1], Wei Chen[3,*], Deyin Guo[2,*], Ronggui Hu[1,*]

[#]These authors contributed equally: Xiangpeng Sheng, Fang Zhu, Hong Peng, Fan Yang, Yi Yang

[*]Correspondence:

Ronggui Hu (coryhu@sibcb.ac.cn); Deyin Guo (guodeyin@mail.sysu.edu.cn); Wei Chen (cwdoctor@163.com)

This PDF file includes:

Materials and Methods

Supplementary Fig. S1 to S7

Materials and Methods

Cell lines

Human embryonic kidney (HEK) cell line 293T and 293FT, human airway epithelial cell line Calu-3, and African green monkey kidney cell line Vero E6 were purchased from National Collection of Authenticated Cell Cultures (Shanghai, China), and cultured at 37 ˚C with 5% CO_2 in Dulbecco's Modified Eagle Medium (DMEM) containing 2 mM L-glutamine and 10% fetal bovine serum (FBS) (Ausbian WS500T). Human ACE2 stably expressing HEK293T cells (293T-hACE2) generated in this study were cultured in DMEM supplemented with 10% FBS. Authentication was confirmed by the suppliers, and by morphology check with light microscopy. All the cell lines used were negative for mycoplasma.

Constructs and plasmids

The plasmid pCMV14-S-3Flag encoding SARS-CoV-2 S glycoprotein with deletion of C-terminal 1-19aa was kindly gifted by Prof. Zhaohui Qian from Peking Union Medical College; pVAX1 vectors carrying genes of several S variants (WT, D614G, Alpha, Beta, Delta) were generously provided by Prof. Dimitri Lavillette, Institute Pasteur of Shanghai, Chinese Academy of Sciences. cDNA of S Omicron was synthesized and then cloned to pVAX1 vector. Genes encoding S protein, its subunits (S1 and S2), or its variants were cloned to pCDNA3.0-Flag for mammalian expression. Gene S or S1 was also cloned to pDEST-32 for yeast expression as a bait of Y2H screening. For a non-cleaved S mutant, coding sequence of RRAR (residues 682-685) at the S1/S2 cleavage site was modified to encode GSAS, as described before [1], and inserted into pCDNA3.0 vector.

A human ORF library was cloned to the pDEST22 backbone for the Y2H screening. The potential candidates identified from the Y2H screening were amplified and cloned into the pGEX-4T1 vector for bacterial expression and purification. CYPA cDNA was modified to bring in a residue substitution (Arg-to-Ala) in residue 55 (R55A) to generate a CypA mutant that loses its isomerase activity. The gene encoding WT or R55A CypA was inserted into pCDNA3.0-HA or pGEX-4T1 for eukaryotic or prokaryotic expression, respectively. Various CypA fragments were also generated by PCR and transferred into pGEX-4T1 for protein purification.

Antibodies and inhibitors

The antibody for CypA (#AF3589) was obtained from R&D systems. Rabbit anti-S1 (# A20136) and anti-S2 (#A20138) polyclonal antibodies and Recombinant CypA protein (#RP00016) were purchased from ABclonal. Anti-Flag (#RLI-01), anti-HA (#RLI-02), anti-GST (#RLI-05), anti-His (#RLI-06), and anti-GFP

(#RLI-09) mouse antibodies were provided by Biolinkedin (Shanghai). Human biotinylated ACE2 protein (#10108-H08H-B) was from Sino Biological. Cyclosporine A (#SC5120) was ordered from Solarbio. Alisporivivr (#HY-12559), NIM811 (#HY-P0025), and TMN355 (HY-107635) were purchased from MCE. Blasticidin (#ant-bl-1) was obtained from InvivoGen.

Yeast two-hybrid (Y2H) screening

GAL4-based yeast two-hybrid system was utilized to screen S1-interacting proteins, as previously described.[2] In brief, yeast Mav203 strain harboring pDEST32-S1 was used to make competent cells. The yeast competent cells were subsequentially transformed with a pDEST22-based human ORF library. Yeast transformants were cultured and selected on SD4 plates (deficient in Leu, Trp, His and Ura) for two weeks. Yeast clones that grew up on SD4 were subjected to expanded culture, and their plasmids were then extracted and sequenced.

Expression and purification of recombinant proteins

Protein expression and purification in *E. coli* had been described before.[3] *E. coli* BL21 (DE3) harboring recombinant plasmids were grown on LB plates containing appropriate antibiotics overnight. A single colony was picked and cultured in 10 mL LB medium for 16 h, and was then expanded to 1 L medium for further culture. When OD600 reached 0.6 ~ 0.8, IPTG (0.1 ~ 0.5 mM) was added to induce expression of the recombinant proteins at 16 ˚C overnight. To purify GST-tagged proteins, bacteria were collected at 5000 rpm for 5 min, and lysed in NETN buffer (50 mM Tris (PH 7.6), 150 mM NaCl, 0.5% NP40, 1 mM EDTA, 2 mM PMSF and 1 mM DTT), and proteins were purified by affinity chromatography using Glutathione Sepharose 4B beads (Cytiva). For His_6-tagged proteins, purification was proceeded with Ni-NTA beads (Qiagen) according to the manufacturer's instruction. Purified proteins were dialyzed overnight in dialysis buffer (20 mM Tris (PH 7.6), 500 mM NaCl, 2 mM DTT and 20 % Glycerol) at 4˚C. To purify trimeric S protein, 293T cells were transfected with S_{GSAS}-expressing plasmids for several days, and culture supernatant was collected for purification, according to a protocol reported before [1]. Protein concentrations were measured by the BCA protein assay, and protein purities were estimated by Coomassie blue staining in SDS-PAGE gels. After flash-freezing in liquid nitrogen, proteins were transferred to -80 ˚C.

GST pull-down (PD) assay

GST or GST-tagged proteins, purified S proteins or spike-expressing cell lysates and Glutathione Sepharose 4B beads (Cytiva) were mixed in total 1 mL NETN buffer (50 mM Tris (PH 7.6), 150 mM NaCl, 0.5% NP40, 1 mM EDTA, 2 mM PMSF and 1 mM DTT) supplemented with protease inhibitor cocktail (Roche

cOmplete™). After incubation at 4˚C for 2 h or overnight, beads were washed for 5 times with NETN buffer, and then prepared in SDS loading buffer for SDS-PAGE.

Co-immunoprecipitation (Co-IP)

Plasmids expressing HA-tagged CypA and Flag-tagged S, S1 or S2 were transfected into HEK293T cells for 24 h. Co-IP buffer containing 50 mM Tris-HCl (PH 7.4), 150 mM NaCl, 1% Triton X-100, 250 U Benzonase™ Nuclease and 1% protease inhibitor cocktail, was utilized to lyse cells at 4 ˚C for 1 h. Cell lysate was centrifuged at 15000 rpm for 10 min to remove debris. The anti-HA antibody-conjugated Sepharose beads were then incubated with the supernatant overnight. The beads were washed with 1 mL Co-IP buffer at 4 ˚C for 10 min with rotation. After washing for five times, beads were collected and boiled in SDS-PAGE loading buffer, and subjected to IB analysis with indicated antibodies.

Gene knockout by CRISPR/Cas9 system

Three guide sequences targeting human *CYPA* gene were designed by online CRISPOR program (http://crispor.tefor.net/):

(1) 5'- CTTGGGCCGCGTCTCCTTTG -3';

(2) 5'- TTCTTCGACATTGCCGTCGA -3';

(3) 5'- ATCCTAAAGCATACGGGTCC -3'.

These target sequences were synthesized and then cloned to lentiCRISPRv2 backbone (Addgene) according to a published protocol.[4] Packaging plasmid psPAX2 (Addgene), envelope plasmid pMD2.G (Addgene) and lentiCRISPRv2 plasmids encoding CypA-sgRNAs were co-transfected into HEK293FT cells with a ratio of 2.5:1.0:3.5. After transfection for 48h, the virus supernatant was collected and filtrated through 0.45 μm filters. 2 mL virus supernatant was mixed with 2 mL fresh complete medium and polybrene (8 μg/mL), and added to target cells in a 6-well plate. After 24 h transduction, cells were cultured in fresh complete medium for further 24 h. Cells were trypsinized and transferred to a 10 cm tissue culture dish in 10 mL medium supplemented with selection antibiotic (InvivoGen) and cultured for 5 more passages. Cells were sorted and seeded into 96-well plates by flow cytometry. Colonies were expanded and subjected to anti-CypA IB analysis for identification of knockout efficiency. Gene sequencing was performed to further verify *CYPA* deletion in the knockout candidates.

Production and purification of SARS-CoV-2 S pseudovirions

S pseudovirus production was performed as published previously with some modifications.[5] psPAX2, pCDH-sfGFP, and a plasmid expressing S without C-terminal cytoplasmic tail (ratio: 4.5:3:3) were co-transfected into HEK293FT cells by using Lipofectamine™ 2000 Reagent (Invitrogene). After 48 h, the virus supernatants were harvested and filtrated with 0.45 µm filters. Pseudovirus purification was carried out with the Lenti-X™ Maxi Purification Kit (TaKaRa) according to the manufacturer's instruction. The purified pseudovirions or raw virus supernatant was aliquoted and stored at -80 ˚C.

Analysis of spike oligomeric status on virions

The oligomeric status of spike on the purified pseudovirions was analyzed based on a previous method with modifications[6]. Briefly, we purified pseudovirions and used 2% Triton X-100 to solubilize pseudovirions. Virus lysate was mixed with native gel sample loading buffer (without SDS/DTT) (Beyotime biotechnology, P0016N) and loaded into native PAGE gels (Tris-Gly, 4-20%) (Beyotime biotechnology, P0469S); the gels were run in 1x SDS running buffer. After running, the PAGE gels were soaked in 1x pre-cold transfer buffer containing 20% methanol for 30 min, and then subjected to membrane transfer and western blot analysis.

S pseudovirus infection assay

HEK293T-hACE2 cells were trypsinized into single cells and incubated with 1 mL media containing S pseudovirions and DMSO or indicated inhibitors, and seeded into 12-well plates. Cells were cultured at 37 ˚C with 5% CO_2. 24 h later, fresh medium was added and cells were further cultured for another 24 h. Cells were trypsinized and subjected to fluorescent microscopic or flow cytometric analysis to determine the percentage of GFP-positive cells. All experiments were performed in triplicates.

Authentic SARS-CoV-2 infection assay

Authentic SARS-CoV-2 infection was performed in BSL-3 facility. Vero E6, Calu-3, or 293T-hACE2 cells were seeded into 48-well plates at $2{*}10^4$ cells per well, overnight. Cells were infected with SARS-CoV-2 virions for 48 h, with DMSO or inhibitors if indicated. After infection, cells or culture supernatants were collected for IB or qRT-PCR analysis. All infections were performed in triplicates.

Plaque assay of authentic SARS-CoV-2

The SARS-CoV-2 plaque assay was carried out as described before.[7] In brief, Vero E6 cells were cultured in 12-well plates with 80% confluence and infected with SARS-CoV-2 at indicated concentrations. After 1 h incubation at 37°C, the supernatants were removed. Cells were then cultured with 1 mL DMEM medium

containing 2% FBS and 0.8% agar (Amresco). Three days later, cells were stained with the staining buffer containing 70% ethanol and 0.5% crystal violet (Sigma-Aldrich) for 24 h. Plaques were counted and analyzed.

Split NanoLuc-based cell-cell fusion assay

The split NanoLuc-based cell-cell fusion assay was designed according to a study published before.[8] The indicated plasmids were transfected into 293FT or 293T-hACE2 cells. 24 h after transfection, 293FT cells expressing LgBit and S and 293T-hACE2 cells expressing HiBit were trypsined and cocultured at a 1:1 ratio for about 5 h. Substrate furimazine was then added to cells, and luminescence intensity was detected and calculated.

Statistical analysis

Data are shown as means±SD. Statistical analysis was performed primarily with GraphPad Prism (GraphPad Software, LLC). Student's *t*-test was conducted for the comparison between two groups. In all assays, *P* values less than 0.05 were considered to be statistically significant. Data from at least three independent experiments were analyzed.

Reference

1 Xiong, X. *et al.* A thermostable, closed SARS-CoV-2 spike protein trimer. *Nat. Struct. Mol. Biol.* **27**, 934-941 (2020).

2 Liu, Z. *et al.* Ubiquitylation of autophagy receptor Optineurin by HACE1 activates selective autophagy for tumor suppression. *Cancer cell* **26**, 106-120 (2014).

3 Sheng, X. *et al.* Bacterial effector NleL promotes enterohemorrhagic E. coli-induced attaching and effacing lesions by ubiquitylating and inactivating JNK. *PLoS Pathog.* **13**, e1006534 (2017).

4 Sanjana, N. E., Shalem, O. & Zhang, F. Improved vectors and genome-wide libraries for CRISPR screening. *Nat. Methods* **11**, 783-784 (2014).

5 Ou, X. *et al.* Characterization of spike glycoprotein of SARS-CoV-2 on virus entry and its immune cross-reactivity with SARS-CoV. *Nat. Commun.* **11**, 1-12 (2020).

6 Song, H. C. *et al.* Synthesis and characterization of a native, oligomeric form of recombinant severe acute respiratory syndrome coronavirus spike glycoprotein. *J. Virol.* **78**, 10328-10335 (2004).

7 Cao, L. *et al.* The adenosine analog prodrug ATV006 is orally bioavailable and has preclinical efficacy against parental SARS-CoV-2 and variants. *Sci. Transl. Med.* **14**, eabm7621 (2022).

8 Dixon, A. S. *et al.* NanoLuc complementation reporter optimized for accurate measurement of protein interactions in cells. *ACS Chem. Biol.* **11**, 400-408 (2016).

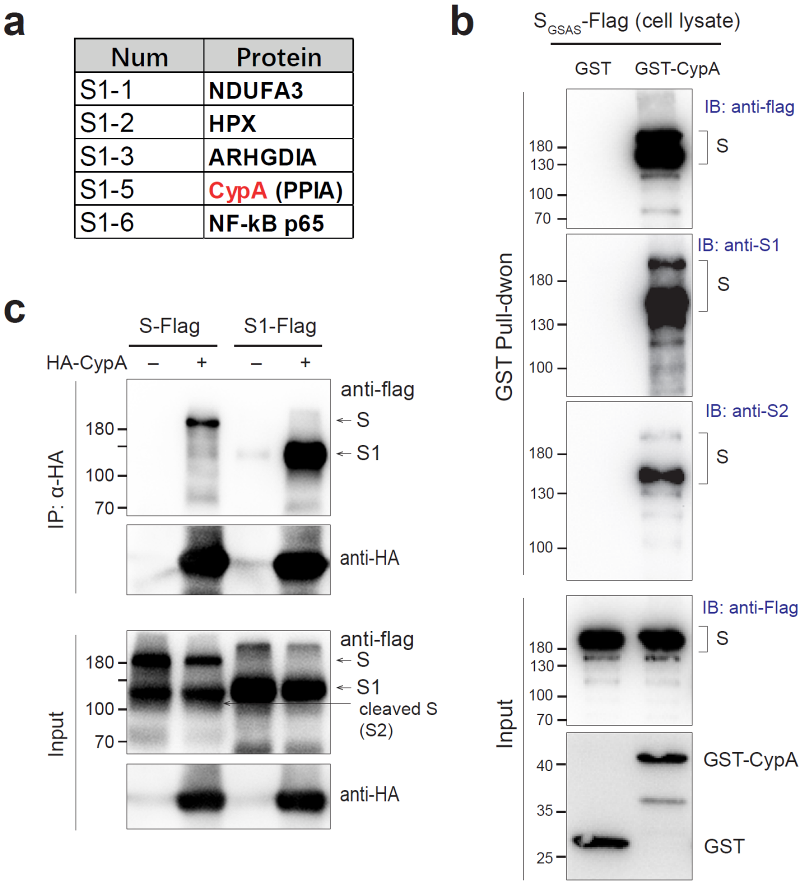

Figure S1. Human CypA binds to SARS-CoV-2 S protein. (a) Potential S-binding candidates identified from a Y2H screen are listed. **(b)** CypA recruits low- and high-glycosylated S. Cell lysate of 293T expressing Flag-S_{GSAS} was incubated with GST or GST-CypA in a GST pull-down assay. IB was then performed with indicated antibodies. **(c)** S and S1 can form complexes with CypA in mammalian cells. Co-expression of C-terminal Flag-tagged S or S1 and HA-tagged CypA in 293T cells, and anti-HA Co-IP was performed 48 h later. IP samples were analyzed by anti-Flag or anti-HA IB. The arrowheads mark the indicated bands.

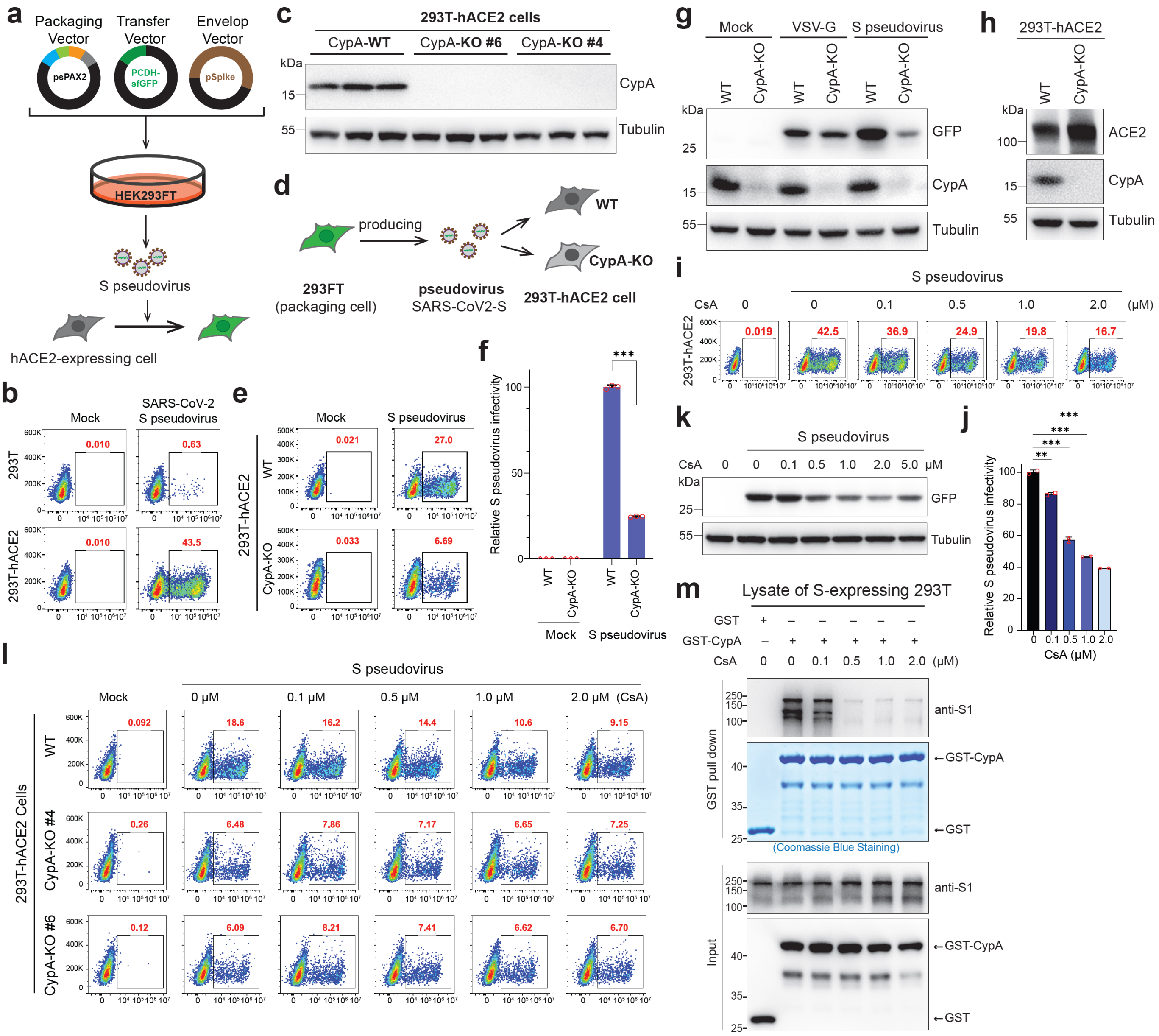

Figure S2. CypA deficiency attenuates the infectivity of S pseudovirus in target cells. (a) S pseudovirus was packaged and generated by a three-plasmid system in 293FT cells. psPAX2, pCDH-sfGFP, and a plasmid expressing SARS-CoV-2 S were co-transfected into 293FT cells. 48 h later, viral supernatant was collected, filtrated, and stored at -80°C. S pseudovirus virions can infect hACE2-positive cells, and the infected cells will express GFP protein. **(b)** SARS-CoV-2 S pseudovirus readily infect hACE2-positive cells. 293T or 293T-hACE2 cells were infected with S pseudovirus for 24 h and subjected to flow cytometric analysis. **(c)** CypA-deficient 293T-hACE2 cells are successfully constructed using the CRISPR-Cas9 system. sgRNA-expressing plasmids were introduced to 293T-hACE2 cells for 48 h, and 10 μg/μL Blasticidin was used to select cells for 72 h. Survival cells were sorted into 96-well plates, one cell for each well. After 2-week growth, single clones were expanded and tested by anti-CypA IB. **(d)** Schematic of the infection in WT or CypA-KO 293T-hACE2 cells by S pseudovirus generated from 293FT cells. **(e, f)** CypA deficiency in 293T-hACE2 attenuates S pseudovirus infection. WT or CypA-KO 293T-hACE2 was incubated with or without S pseudovirus. After

24 h, cells were analyzed by flow cytometry. Shown in (**e**) are flow cytometric plots with the proportions of GFP-positive cells. Comparison of percentages of GFP-positive cells are present in (**f**). **(g)** CypA deficiency in 293T-hACE2 robustly blocks the infection of S pseudovirus, but only weakly disrupts VSV-G pseudovirus infection. Purified S or VSV-G pseudovirus was utilized to infect WT or CypA-KO 293T-hACE2 cells for 24 h. Samples were then analyzed by IB with anti-GFP antibody. **(h)** Deletion of CypA cannot decrease ACE2 protein level in host cells. **(i, j)** CsA exhibits a dose-dependent inhibitory effect on S pseudovirus infection in 293T-hACE2 cells. Equal amounts of S pseudovirus and 293T-hACE2 cells were mixed with different concentrations of CsA, and cells were seeded in 12-well plates for 24 h. The percentage of GFP-positive cells was examined by flow cytometry analysis. Shown in (**i**) are flow cytometric scatter plots with proportions of GFP-positive cells. Comparisons of the percentage of GFP-positive cells are shown in (**j**) (n=2). **(k)** CsA prevents the infection of S pseudovirus in hACE2-expressing cells. 293T-hACE2 cells were infected by S pseudovirus, with synchronous treatment of different concentrations of CsA. 24h later, cells were lysed and analyzed by anti-GFP IB. **(l)** Knockout of CypA in 293T-hACE2 abolishes the inhibitory roles of CsA on viral infection. WT or CypA-KO 293T-hACE2 cells were infected by SARS-CoV-2 pseudovirus and incubated with different concentrations of CsA. Shown are the flow cytometric data. **(m)** CsA efficiently disrupts S-CypA interaction in a dose-dependent manner. Cell lysate of S-expressing 293T cells, GST-CypA, and different concentrations of CsA were incubated in GST pull-down buffer, and glutathione (GSH) agarose beads were used to capture GST-CypA. Data are present as mean ± SD, $^{**}P < 0.01$, $^{***}P < 0.001$. (Student's *t*-test, biological triplicates of infected cells (n = 3)).

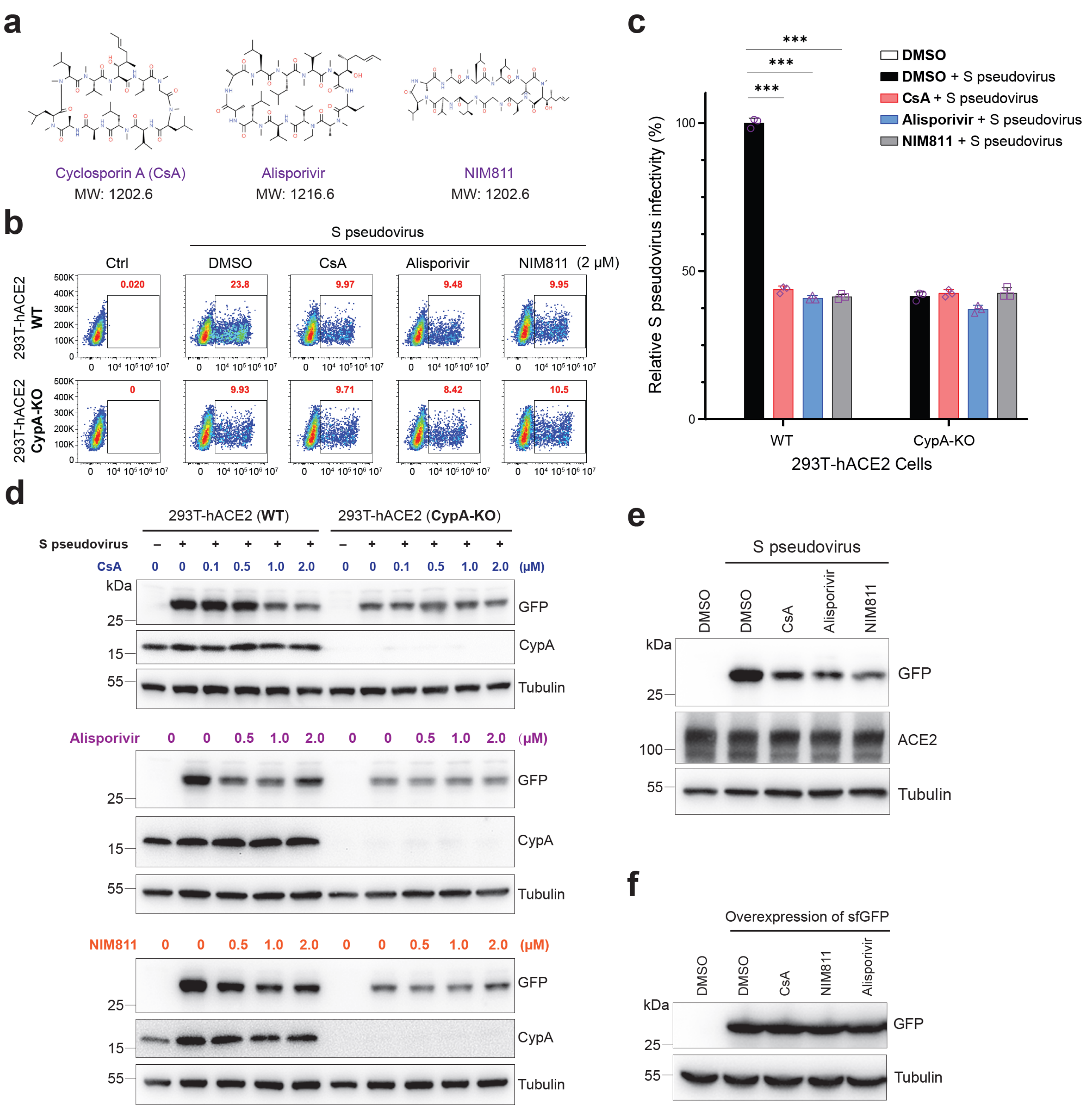

Figure S3. CsA derivatives also readily impair the infectivity of S pseudovirus. (a) Structural formulas of CypA inhibitors, including CsA, and its derivatives (NIM811 and alisporivir). These structural formulas are from ChemSpider. **(b)** Cyclic peptide inhibitors of CypA prevent S pseudovirus infection by targeting CypA. WT or CypA-KO cells incubated with SARS-CoV-2 S pseudovirus and CypA inhibitors (2 μM) for 24 hours. Cells were analyzed by flow cytometry. **(c)** Quantitative analysis of the relative infectivity in (b). **(d)** CsA and its derivatives did not inhibit S pseudovirus infection in CypA-deficient cells. WT or CypA-KO cells were incubated with S pseudovirus and different concentrations of inhibitors for 24 h, and cell samples were analyzed by western blot. **(e)** CsA and its analogs show inhibitory roles on S pseudovirus infection but not ACE2 expression. 293T-hACE2, that were incubated with indicated CypA inhibitors and S pseudovirus for 24 h, were lysed in SDS loading buffer and analyzed by anti-GFP or anti-ACE2 IB. (**f**) CypA inhibitors have no impact on GFP expression in transfection assay.

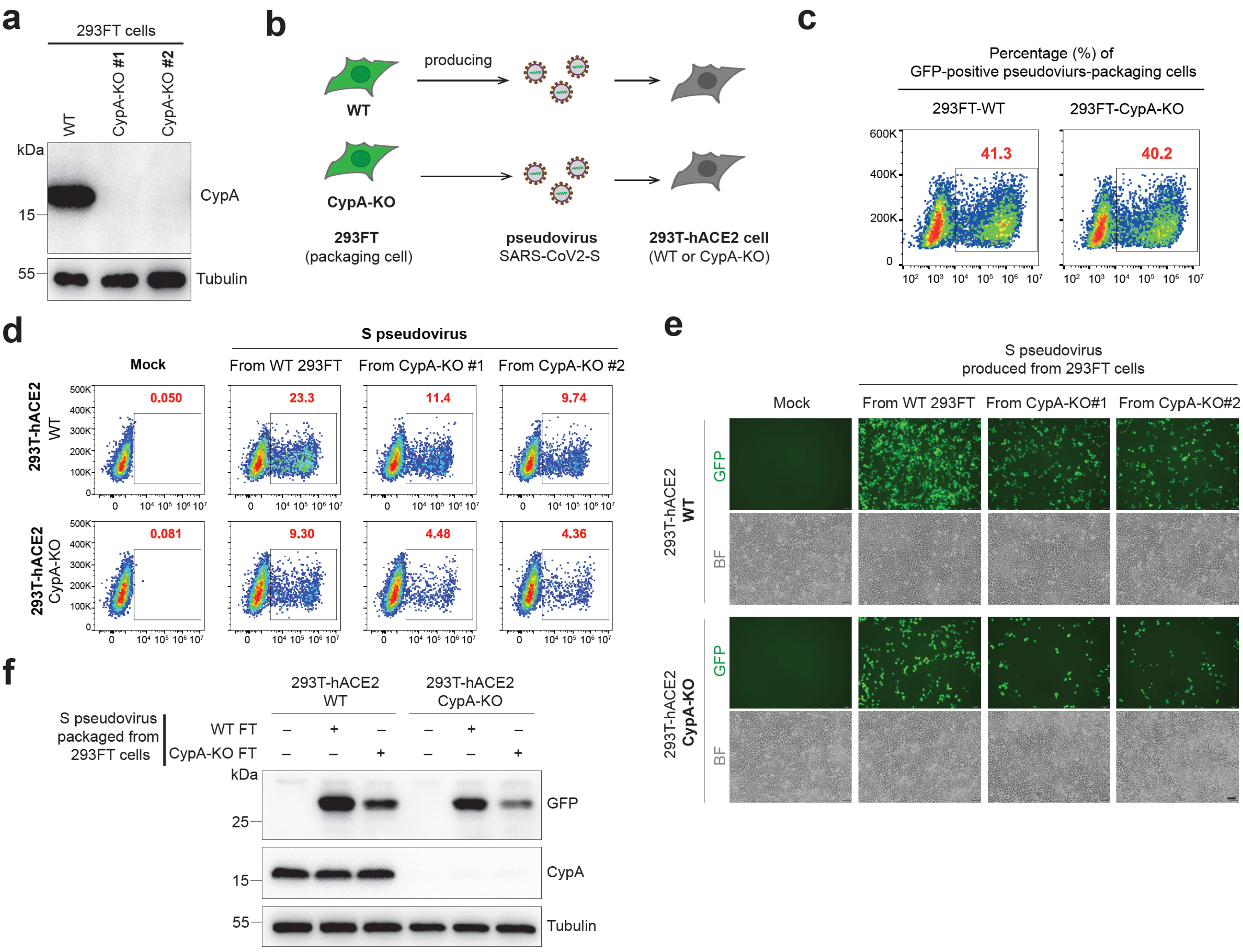

Figure S4. Deficiency of CypA in packaging cells impairs viral infectivity. (a) Two CypA-KO 293FT cell lines have been constructed. Cell lines were constructed by the CRISPR-Cas9 system. Shown is anti-CypA IB result of the cell lines. **(b)** Schematic of an infection strategy that SARS-CoV-2 pseudovirus generated from WT or CypA-KO 293FT packaging cells was utilized to infect WT or CypA-KO 293T-hACE2 cells. **(c)** WT and CypA-KO 293FT packaging cells show comparable transfection efficiencies. Packaging plasmids were transfected into 293FT cell lines for 48 h, and the GFP-positive cells were detected by flow cytometry. **(d)** Removal of CypA in 293FT packaging cells impairs infectivity of the pseudovirus they produced. WT or CypA-KO 293T-hACE2 cells were infected by S pseudovirus produced from WT, or CypA-KO 293FT cells. **(e)** S pseudovirus generated from CypA-KO 293FT cells has lower infectivity. 293T-hACE2 was infected by viruses produced from different packaging cell lines. After 24h, 293T-hACE2 were imaged by microscopy. Shown are the GFP and BF images. Scale bars, 50 μm. **(f)** Packaging cells deficient of CypA exhibit decreased capacity to generate active virions. 293T-hACE2 cells were infected by viruses produced from different cell lines, and then subjected to anti-GFP western blot.

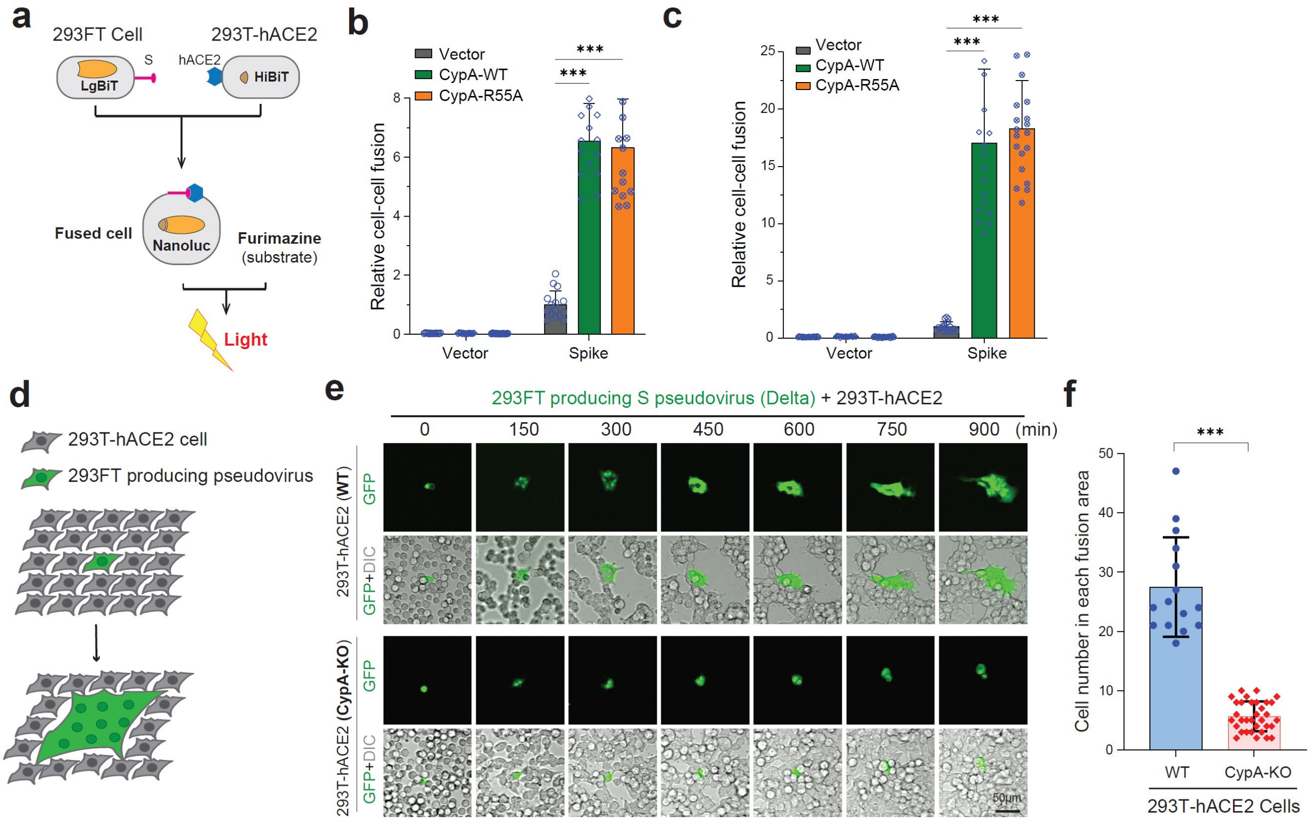

Figure S5. CypA facilitates S-mediated cell-cell membrane fusion. (a) Schematic illustration of the cell-cell fusion reporter assay using split-NanoLuc (LgBiT/HiBiT). **(b)** Quantitative analysis of the relative levels of cell fusion in **Fig. 1l**. Cell fusion areas were measured via Photoshop, and relative cell fusion levels were calculated and shown. Data are present as mean ± SD, $^{***}P < 0.001$. (Student's *t*-test, n = 15). **(c)** Quantitative analysis of the relative levels of syncytia formation in **Fig. 1m**. Data are present as mean ± SD, $^{***}P < 0.001$. (Student's *t*-test, n = 20). **(d)** Schematic illustration of the dynamic pseudovirus-based cell-cell fusion assay. A small number of S pseudovirus-packaging cells (GFP-positive) were mixed with large numbers of 293T-hACE2 cells, and cultured overnight. Cell fusion was observed by microscopy. **(e)** CypA knockout restricts pseudovirus-mediated cell-cell fusion and transmission. Fusion assay was performed as described in (**d**), and then analyzed by the real-time live-cell imaging technology for 15 h since seeding. Scale bars, 50 μm. **(f)** Quantitative analysis of nucleus numbers in syncytia in **Fig. 1o**. Data are presented as mean ± SD, $^{***}P < 0.001$. (Student's *t*-test, n ⩾15).

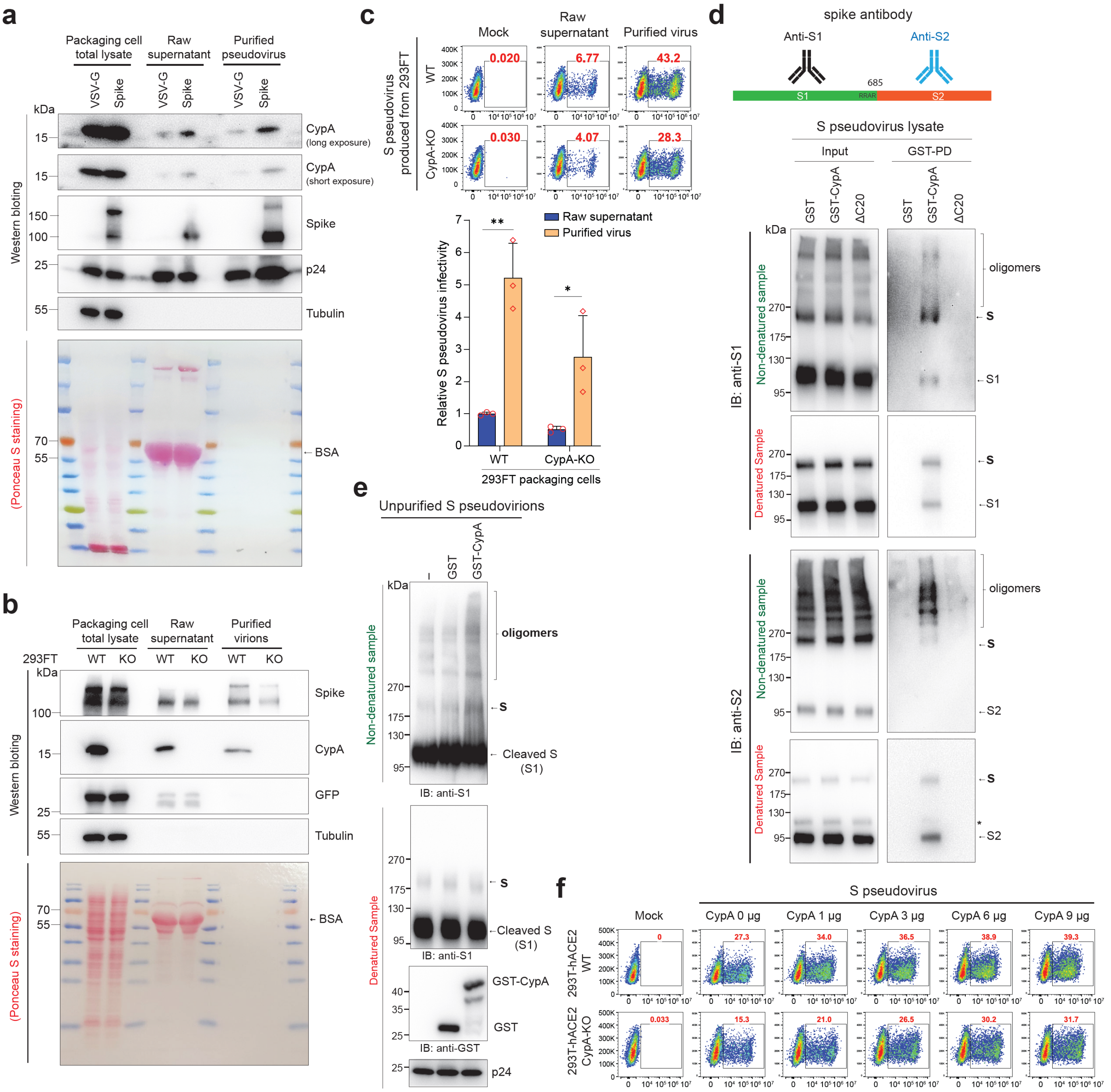

Figure S6. Human CypA interacts with SARS-CoV-2 S on virions and promotes S oligomerization. (a) Purified S pseudovirus captured much more CypA than VSV-G pseudovirus. S or VSV-G pseudovirus was packaged in WT 293FT. Viral supernatant was collected, filtrated by 0.45 μm filters, and purified. Packaging cell lysates, raw viral supernatant, and purified virions were analyzed by IB with indicated antibodies or by ponceau S staining. **(b)** Purified S pseudoviruses recruit endogenous CypA from human cells. S pseudovirus was packaged in WT or CypA-deficient 293FT. **(c)** Purification process in (**b**) leads to S pseudovirus enrichment. The enrichment of virions after purification was estimated by the infection assay in 293T-hACE2 cells. The top panel shows the flow cytometric plots with GFP gating; the bottom panel is a quantitative analysis of purified virions. **(d)** CypA can interact with S monomers and oligomers of virions. S pseudovirus

was solubilized in Triton X-100 buffer and subjected to a pull-down assay with GSH beads and GST, GST-CypA, or GST-CypA-△C20. After washing, proteins recruited to GSH beads were eluted by elution buffer (20 mM GSH), and used to prepare non-denatured and denatured samples for IB analysis. The upper panel shows a diagram for the specific antibody recognizing S1 or S2 domain. The lower panel is IB results. **(e)** Recombinant CypA promotes S oligomerization on the virions surface. Unpurified viral supernatant was incubated with GST or GST-CypA at 37°C for one hour, and was lysed and subjected to anti-S1 IB analysis. **(f)** Incubation of CypA recombinant protein with S pseudovirus promoted viral infectivity in 293T-hACE2 cells. S pseudoviruses incubated with different amounts of CypA recombinant protein were utilized to infect WT or CypA-KO 293T-hACE2 cells for 24 hours. 293T-hACE2 cells were then analyzed by flow cytometry.

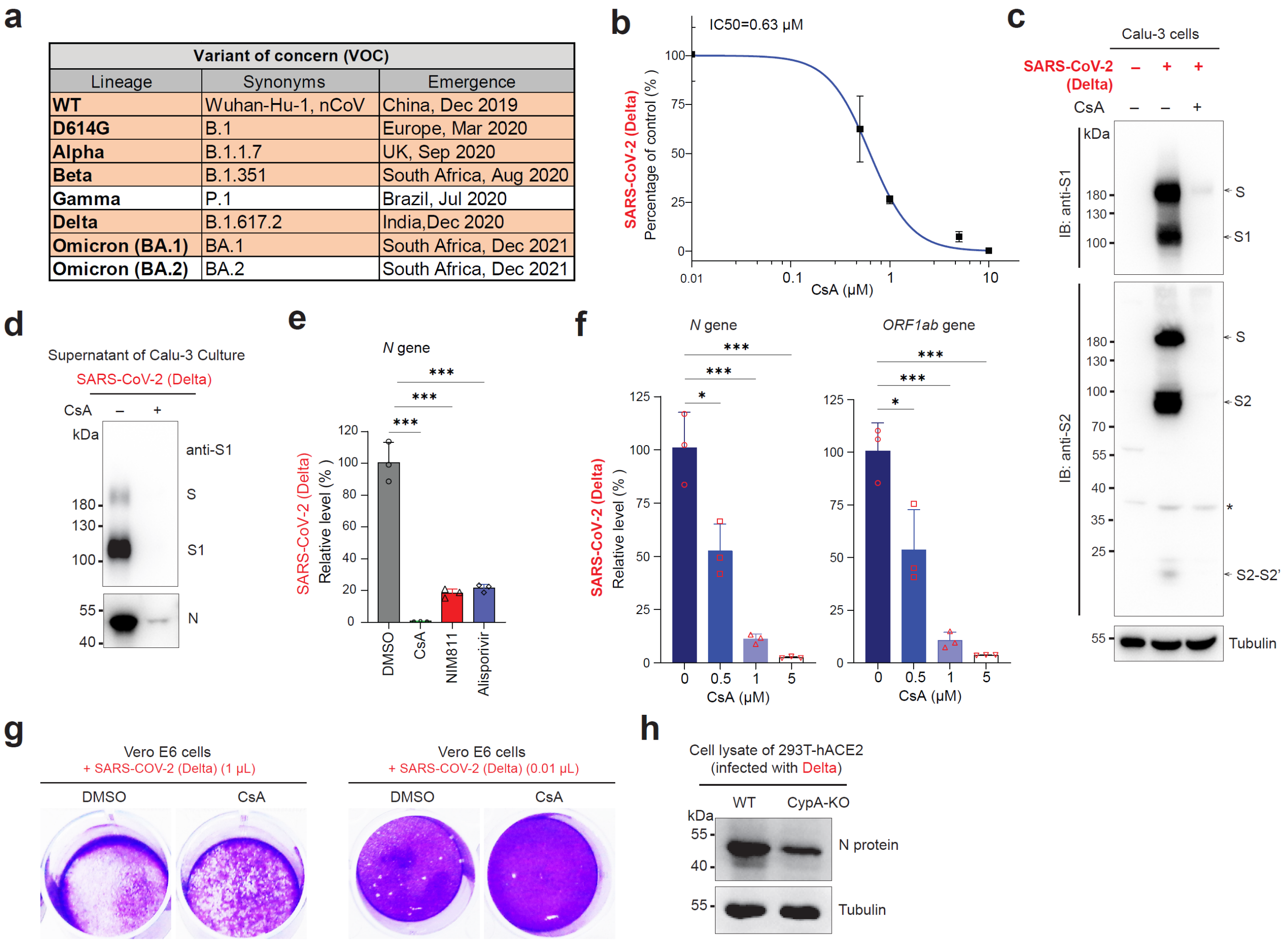

Variant of concern (VOC)		
Lineage	Synonyms	Emergence
**WT**	Wuhan-Hu-1, nCoV	China, Dec 2019
**D614G**	B.1	Europe, Mar 2020
**Alpha**	B.1.1.7	UK, Sep 2020
**Beta**	B.1.351	South Africa, Aug 2020
**Gamma**	P.1	Brazil, Jul 2020
**Delta**	B.1.617.2	India,Dec 2020
**Omicron (BA.1)**	BA.1	South Africa, Dec 2021
**Omicron (BA.2)**	BA.2	South Africa, Dec 2021

Figure S7. Targeting CypA prevents the infection of SARS-CoV-2 variants. (a) A list of several SARS-CoV-2 variants of concern. **(b)** Dose-response analysis of inhibitory effects of CsA on SARS-CoV-2 Delta infection in Vero E3 cells. Data represent mean ± SD of biological triplicates. **(c)** CsA efficiently blocks SARS-CoV-2 Delta infection in Calu-3 cells. Calu-3 cells were incubated with SARS-CoV-2 Delta and 2.0 μM CsA. 48 h later, cells were harvested and analyzed by IB. The asterisk indicates a non-specific band. **(d)** CsA blocks the proliferation of SARS-CoV-2 Delta in Calu-3 cells. Calu-3 treated with or without CsA was infected by Delta variant. After infection, the culture supernatant was analyzed by anti-S1 or anti-N IB. **(e)** Cyclic peptide inhibitors of CypA significantly inhibit infection of SARS-CoV-2 Delta. Vero E6 cells were incubated with Delta variant and indicated inhibitors. After infection, supernatant of cells was harvested and analyzed by qPCR of viral gene *N*. **(f)** CsA prevents SARS-CoV-2 Delta infection in a dose-dependent manner. Vero E6 cells infected by Delta variant were treated with different concentrations of CsA. After 48 h, culture medium was collected and subjected to qPCR analysis of viral gene *N* or *ORF1ab*. **(g)** Plaque formation of SARS-CoV-2 Delta in Vero E6 cells was strongly suppressed by CsA. Cells were incubated with CsA and different concentrations of SARS-CoV-2 Delta in 12-well plates. After several days, plaques were formed. Cells were fixed and stained with 0.5% (w/v) crystal violet. **(h)** CypA deficiency in 293T-hACE2 impairs SARS-CoV-2 Delta infection. WT and CypA-KO 293T-hACE2 cells were infected by Delta variant. 48 h later, cells were lysed and analyzed by anti-N IB analysis.
